# Development of synovial sarcoma organoids exhibiting ferroptosis resistance despite low malic enzyme 1 expression

**DOI:** 10.1038/s41598-025-32030-w

**Published:** 2025-12-21

**Authors:** Toru Wakamatsu, Yoshiki Yamada, Keiichi Yoshida, Yukiko Matsuoka, Satoru Sasagawa, Noriko Nagamine, Kazuko Shizuma, Rie Suzuki, Hironari Tamiya, Yoshinori Imura, Sho Nakai, Seiji Okada, Toshinari Yagi, Ken-ichi Yoshida, Shigeki Kakunaga, Yoshihiro Yui, Satoshi Takenaka

**Affiliations:** 1 Department of Musculoskeletal Oncology Service, Osaka International Cancer Institute, 3-1-69 Otemae, Chuo-Ku, Osaka, 541-8567 Japan; 2 Department of Orthopaedic Surgery, Osaka University Graduate School of Medicine, 2-2 Yamadaoka, Suita, Osaka 565-0871 Japan; 3 Next-Generation Precision Medicine Research Center, Osaka International Cancer Institute, 3-1-69 Otemae, Chuo-Ku, Osaka, 541-8567 Japan; 4 Molecular Biology Laboratory, Research Institute, Nozaki Tokushukai Hospital, Tanigawa 2-10-50, Daito, Osaka 574-0074 Japan; 5 Department of Cancer Immunotherapy, Research Center, Osaka International Cancer Institute, 3-1-69 Otemae, Chuo-Ku, Osaka, 541-8567 Japan; 6 Department of Diagnostic Pathology and Cytology, Osaka International Cancer Institute, 3-1-69 Otemae, Chuo-Ku, Osaka, 541-8567 Japan; 7 Sarcoma Treatment Laboratory, Research Institute, Nozaki Tokushukai Hospital, Tanigawa 2-10-50, Daito, Osaka 574-0074 Japan

**Keywords:** Synovial sarcoma, Organoid, SS18-SSX, Ferroptosis, Malic enzyme 1 (*ME1*), Cancer, Cell biology, Oncology

## Abstract

Although several studies have investigated synovial sarcoma using established cell lines and mouse models, effective treatments remain limited. We treated five patients with synovial sarcoma at our institute between 2022 and 2023, and resected tumor specimens were cultured using a modified air–liquid interface organoid method. After serial passaging and xenografting into NOD-scid IL2Rgnull (NSG) mice, patient-derived organoid lines were successfully established in three of five cases. These organoids exhibited stable propagation, tumorigenicity, and retained histological and genetic features of the original tumors, including *SS18–SSX* fusion confirmed by polymerase chain reaction and Sanger sequencing. We then evaluated the organoids’ response to a ferroptosis-inducing agent previously reported to be effective in synovial sarcoma. Despite low malic enzyme 1 (*ME1*) expression, which has been associated with ferroptosis sensitivity, the established organoids were resistant to treatment. This unexpected result suggests additional mechanisms of resistance and highlights the complexity of ferroptosis regulation in synovial sarcoma. Our findings demonstrate the feasibility of establishing synovial sarcoma organoids using a modified culture method and suggest their potential as preclinical models for drug screening and mechanistic studies. These models provide a valuable resource for the development of novel therapeutic strategies for this rare and aggressive tumor.

**Supplementary Information:**

The online version contains supplementary material available at 10.1038/s41598-025-32030-w.

## Introduction

Synovial sarcoma (SS) is a malignant soft-tissue tumor characterized by a recurrent chromosomal translocation t(X;18) (X; 18)(p11. 2; q11. 2), leading to the formation of the pathogenic fusion protein, SS18-SSX^1–3^. Approximately two-thirds of synovial SS arise in the deep soft tissues of the lower and upper extremities, often in periarticular regions^1,4,5^. SS can also occur in the trunk and head and neck regions. Clinically, SS typically present as a gradually enlarging mass associated with pain. The disease progresses insidiously, with an initial slow growth phase. Due to its compact and localized nature, SS may be misdiagnosed as a benign lesion based on clinical examination and imaging findings^6,7^. Although SS can develop at any age, its incidence is equally distributed between males and females. A significant proportion of cases occur in adolescents and young adults, with over 70% of diagnoses made before the age of 50^7–9^.

The molecular pathogenesis of SS18-SSX has gradually been elucidated^10,11^. The fusion protein is considered to lead to aberrant behavior in the SWItch/sucrose non-fermentable (SWI/SNF) complex, also known as the BAF complex, or the polycomb repressive complexes 1 and 2 (PRC1 and PRC2)^12–14^. However, there are currently no effective treatments based on this mechanism, and patients with SS have received the same treatment, doxorubicin-based chemotherapy for decades. The treatment options include pazopanib, trabectedin, or eribulin^15–17^.

To treat SS more effectively, bioresources mimicking SS tumors in the human body are needed. Several conventional SS cell lines and a mouse model of SS are available^18–21^. However, no treatment has yet been developed that sufficiently improves the prognosis. Therefore, we focused on the three-dimensional (3D) organoid culture methods that have been widely used in various types of cancer, and established several organoids with sarcoma, including epithelioid sarcoma and malignant giant cell tumors of bone, using the air–liquid interface (ALI) organoid culture method^22–27^. The organoids of these sarcomas showed characteristics similar to those of the original tumors^26,27^. Regarding 3D culture in SS, research on spheroid culture, which is controversial because it is distinct from organoid culture, has been extensively conducted^28–31^. However, there have been no reports on the establishment of a stable, usable organoid from human SS.

The purpose of this study is to establish SS organoids, analyze their characteristics and evaluate their similarity to the original tumors. Additionally, this study also aimed to investigate differences in drug responses using the established SS organoids.

## Results

### Establishment of three organoids from human patients with SS using the ALI organoid culture method

**Fig. 1:**
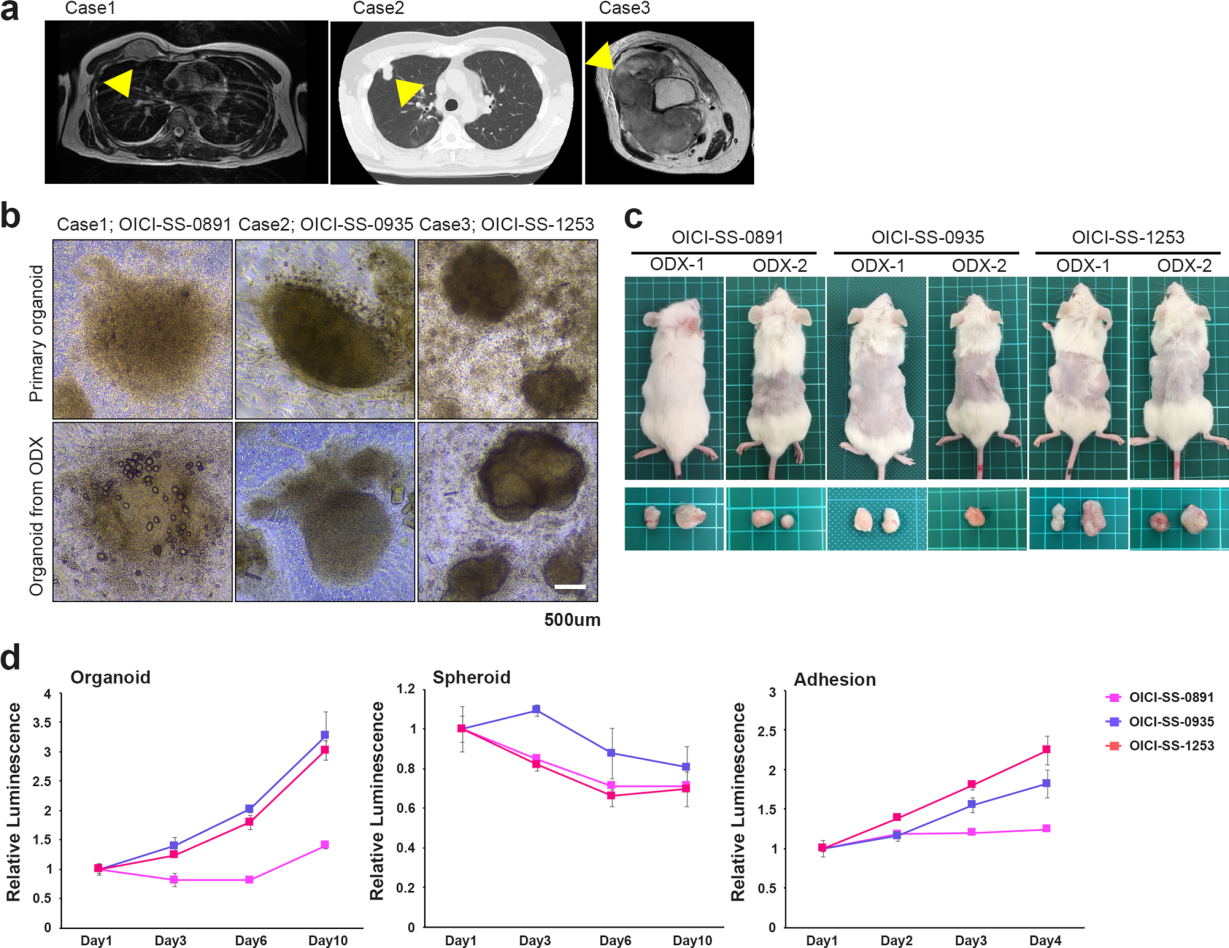
Establishment of PDO and ODX models of three SS cases. (**a**) Clinical images of the SS tumor of three cases. Left (Case 1): T2-weighted axial image of MRI. The tumor was localized in the right chest wall and was a metastatic lesion. Middle (Case 2): A chest CT scan was presented. The tumor was a metastasis in the right lung. Right (Case 3): T2-weighted axial image of MRI. The tumor was localized in the right thigh and the primary lesion. (**b**) The morphology of OICI-SS-0891, OICI-SS-0935, and OICI-SS-1253 organoids under ALI organoid culture by phase-contrast microscopy. The upper panel showed the culture of the primary tumor, while the lower panel presented images of the culture re-established from ODX tumor. (**c**) Developed tumors in NSG mice of ODX-1 and ODX-2 from PDO of SS. (**d**) Relative SS cell viability in organoids or spheroid culture from day1 to 10. Relative SS cell viability on adhesion culture from day 1 to 4. The experiments were performed using ODX-derived organoids established after two serial xenografts in NSG mice. ALI, air–liquid interface; ODX, organoid-derived xenograft; PDO, patient-derived organoids; SS, synovial sarcoma.

We examined ALI organoid cultures in five cases and succeeded in three of these cases. The clinical and experimental information of the examined cases are presented in Table 1. The established organoid lines were named OICI-SS-0891, OICI-SS-0935, and OICI-SS-1253 (Fig. 1a,b, and Table 1). The cells showed stable growth over time in serial passages. The organoid morphology was altered to a spherical shape, with small cell clumps and scattered cells (Fig. 1b). Spindle-shaped cell proliferation was also detected at the periphery of the organoids (Fig. 1b). Regarding the tumorigenicity of SS organoids in NOD-scid IL2Rgnull (NSG) mice, more than 8 months were required to develop tumors in OICI-SS-0891 and OICI-SS-0935; however, tumors were detected within 2–3 months in OICI-SS-1253 mice (Fig. 1c and Table 1). The tumor sizes were as follows (maximum diameter): OICI-SS-0891 (1.1, 1.5 cm), OICI-SS-0935 (1.1, 1.2 cm), OICI-SS-1253 (1.5, 2.0 cm). We cultured the resected ODX from mice using the ALI organoid method or transplanted them into another mouse (Fig. 1b,c). The tumor sizes of transplanted tumors were as follows (maximum diameter): OICI-SS-0891 (1.0, 0.8 cm), OICI-SS-0935 (1.2 cm), OICI-SS-1253 (1.1, 1.4 cm). The size of the OICI-SS-0935 and OICI-SS-1253 organoids increased over a short period and their viability increased by approximately three-fold in 10 days (Fig. 1d). OICI-SS-0891 organoids slowly increased in size and viability (1.4-fold in 10 days) compared with the other SS organoids (Fig. 1d). All organoid models were serially passaged more than four times through patient-derived xenograft (PDX) establishment. OICI-SS-0891 and OICI-SS-0935 were passaged twice before xenografting, whereas OICI-SS-1253 was transplanted directly from the primary culture. No apparent difference in proliferative potential was observed between primary and ODX-derived organoids based on empirical observations. For all experiments, organoids were used after two serial xenografts in NSG mice, which we define as the established ODX-derived models. These SS organoids were capable of forming both spheroid and adherent cultures. However, under spheroid culture conditions, no significant proliferation was observed, whereas adherent cultures demonstrated a growth rate trend similar to that of SS organoids (Fig. 1d).

**Table 1:** Summary of the clinical information of patients with SS and organoid establishment details in this study.

Clinical and experimental information of examined cases in synovial sarcoma										
Case	Organoid-ID	Age/Gender	Tumor location of organoid establishment/Size	Primary/Reccurence/Metastasis	Pretreatment	Outcome	Histological Type	Fusion Type	Time to tumor formation (about 1 cm^3^)	Organoid establishment Success/Failuer
1	OICI-SS-0891	43/F	Right pectoralis major muscle/3.3 cm	Reccurence	DOX + IFM, Pazopanib Clinical Trial drug, Trabectedin	AWD	Monophasic	Type1	8–9 month	Success
2	OICI-SS-0935	49/M	Right lung/2.4 cm	Metastasis	DOX + IFM, HD-IFM, Pazopanib	DOD	Monophasic	Type1	8.5–12 months	Success
3	OICI-SS-1253	66/F	Right thigh/10.5 cm	Primary	–	NED	Biphasic	Type1	2–2.5 months	Success
4	–	68/M	Right forearm/3.3 cm	Primary	–	NED	Monophasic	Type1	–	Failuer
5	–	55/M	Right thigh/4.0 cm	Primary	–	NED	Monophasic	Unknown	–	Failuer

M, male; F, female; DOX, doxorubicin; IFM, ifosfamide; NED, no evidence of disease; AWD, alive with disease; DOD, dead of disease.

### Morphologic characteristics of SS organoids

**Fig. 2:**
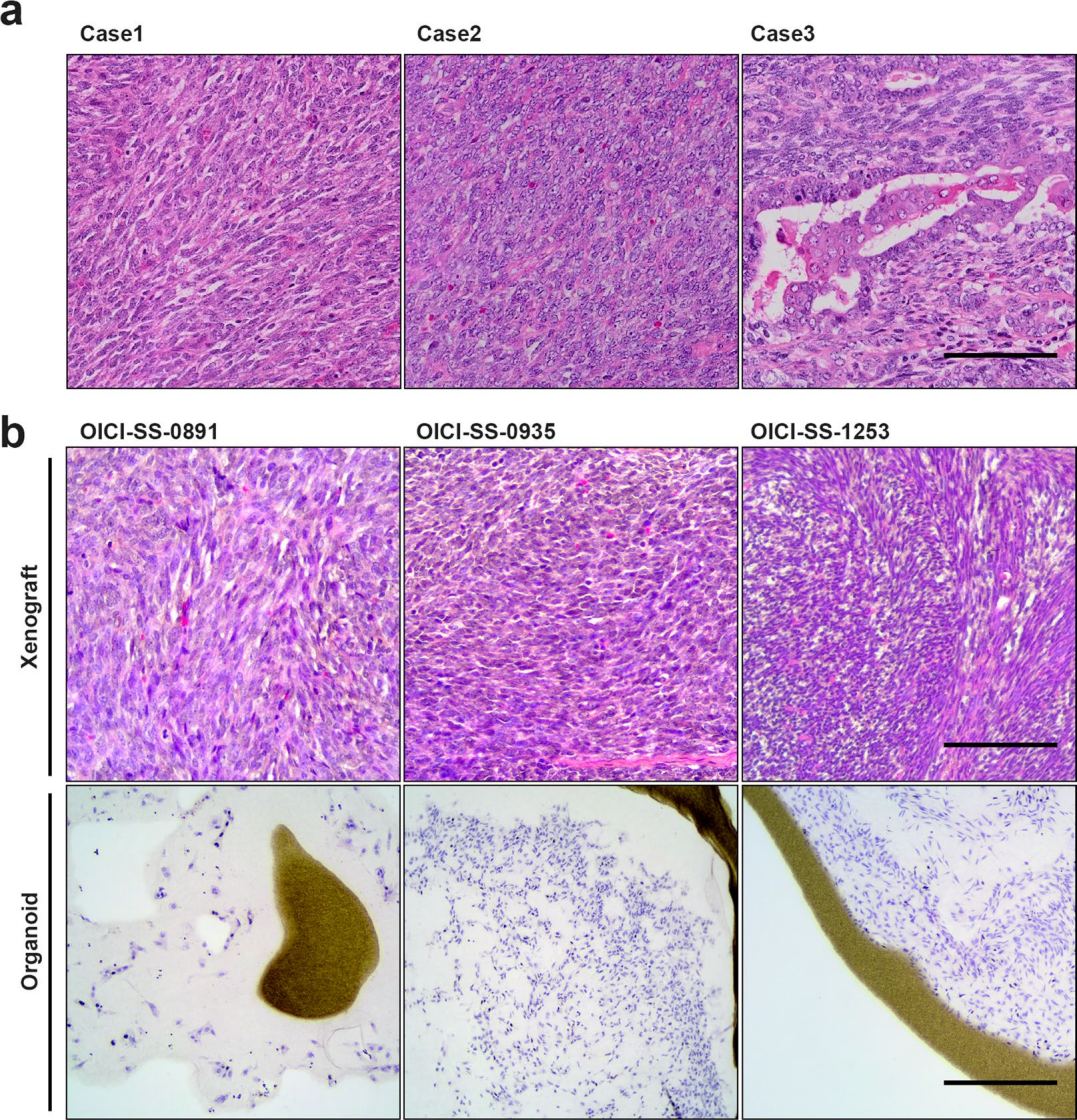
Comparing the microscopic features of the patient’s original SS tumors, and ODX of OICI-SS-0891, OICI-SS-0935, and OICI-SS-1253. (**a**) Histological appearance of the original tumor with H&E staining in three SS cases. (**b**) Histological appearance of the xenografted tumors or cultured SS organoids with H&E staining. The experiments were performed using ODX and ODX-derived organoids established after two serial xenografts in NSG mice. Scale bars: 100 μm. ODX, organoid-derived xenograft; SS, synovial sarcoma.

The pathologist of our institute diagnosed case 1 and 2 as monophasic, and case 3 as biphasic SS, using with hematoxylin and eosin (H&E) staining (Fig. 2a). Histologically, case 1 and 2 showed that uniform tumor cells with polygonal-to spindle-shaped nuclei densely proliferated in an arrangement partitioned by abundant, delicate fibrous vascular stroma. In case 3, there were regions where uniform spindle-shaped and epithelioid cells proliferated in a tubular papillary pattern. To compare the morphological characteristics, we performed H&E staining on the ODX of SS. In OICI-SS-0891 and OICI-0935 cells, ODXs demonstrated a histological appearance resembling that of the original tumor (Fig. 2b). In contrast, ODX from OICI-SS-1253 showed a monophasic rather than biphasic pattern (Fig. 2b). The morphological characteristics of the SS organoids showed a monophasic pattern and the OICI-SS-0891 organoid exhibited a lower cell density than the other two organoids (Fig. 2b). These data demonstrate that the morphological characteristics of OICI-SS-0891 and OICI-SS-0935 were mostly preserved, but OICI-SS-1253 partially preserved its original characteristics during organoid culture and xenograft formation.

### Some SS organoids required the fusion gene, *SS18-SSX,* for their proliferation

**Fig. 3:**
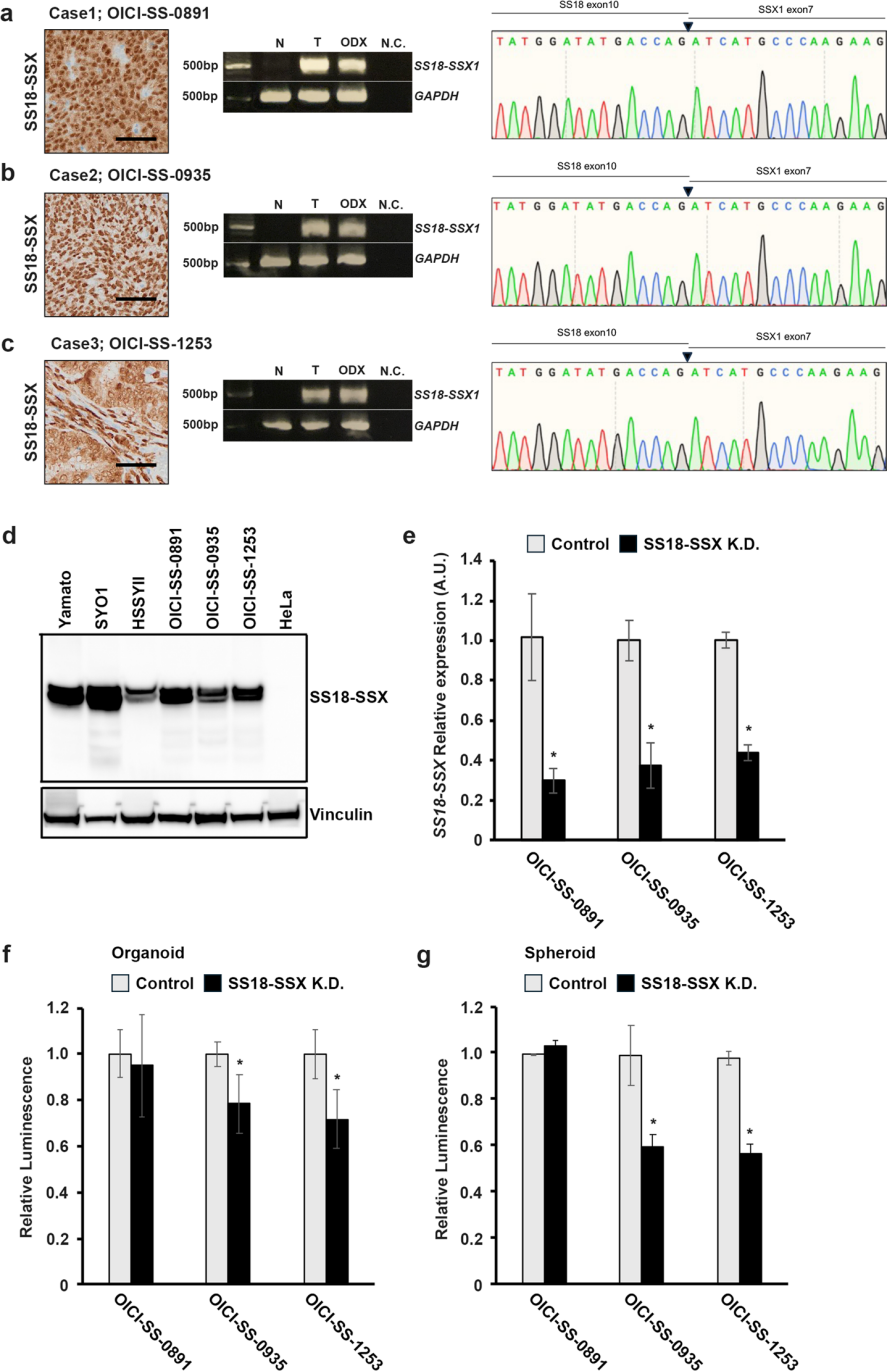
Analysis of the SS18-SSX fusion gene in SS organoids. (**a**–**c**) Left: Immunohistochemical reactivity in the original SS tumors of Case1-3. Tumor cells were diffusely positive for SS18-SSX. Middle: *SS18-SSX* cDNA was identified by PCR in both original tumors and ODX. Right: Sanger sequencing revealed the *SS18-SSX* (Type 1) breakpoint in three cases of SS ODX. Scale bars: 50 μm. (**d**) Fusion protein of SS18-SSX was detected in three SS ODX with western blotting. Yamato, SYO1, and HSSYⅡ were used for positive control. (**e**) qPCR analysis demonstrated the knockdown of the *SS18-SSX* fusion gene using shRNA in three SS organoid lines. This experiment was conducted under spheroid culture conditions. (**f**, **g**) The changes in cell viability following knockdown of the *SS18-SSX* fusion gene under either organoid (**f**) or spheroid (**g**) culture conditions were demonstrated in three SS lines. The experiments in (**e**–**g**) were performed using ODX-derived organoids established after two serial xenografts in NSG mice. ODX, organoid-derived xenograft; SS, synovial sarcoma.

SS is characterized by the fusion gene, *SS18-SSX*. In all three cases in this study, primary tumors expressed SS18-SSX with immunohistology (Fig. 3a,b,c). We verified the fusion gene expression using reverse transcription polymerase chain reaction (RT-PCR) and Sanger sequencing, and this expression was maintained during organoid culture and in the xenograft (Fig. 3a,b,c). All three patients harbored *SS18-SSX* type1 (*SS18-SSX1*). Immunoblotting analysis also demonstrated that established ODX had fusion protein, SS18-SSX similar to commonly used SS cell lines, including Yamato-SS, SYO1, and HSSYⅡ (Fig. 3d). Several studies have reported that the *SS18-SSX* fusion gene is involved in tumor growth and progression by activating transcription, reorganizing chromatin, controlling cell cycle, and inhibiting apoptosis. Therefore, we investigated whether the *SS18-SSX* fusion gene similarly contributed to tumor growth in SS organoids. Knockdown of *SS18-SSX* moderately suppressed proliferation in two out of three synovial sarcoma organoids, with a more pronounced inhibitory effect observed in the spheroids. (Fig. 3e,f,g). In OICI-SS-0891 cells, knockdown of the fusion gene did not suppress proliferation in either organoids or spheroids (Fig. 3e,f,g). These results indicate that the growth of SS organoids required the *SS18-SSX* fusion gene in some cases, but some SS cells proliferated independently of the fusion gene.

### Transcriptome profiling in SS organoids

**Fig. 4:**
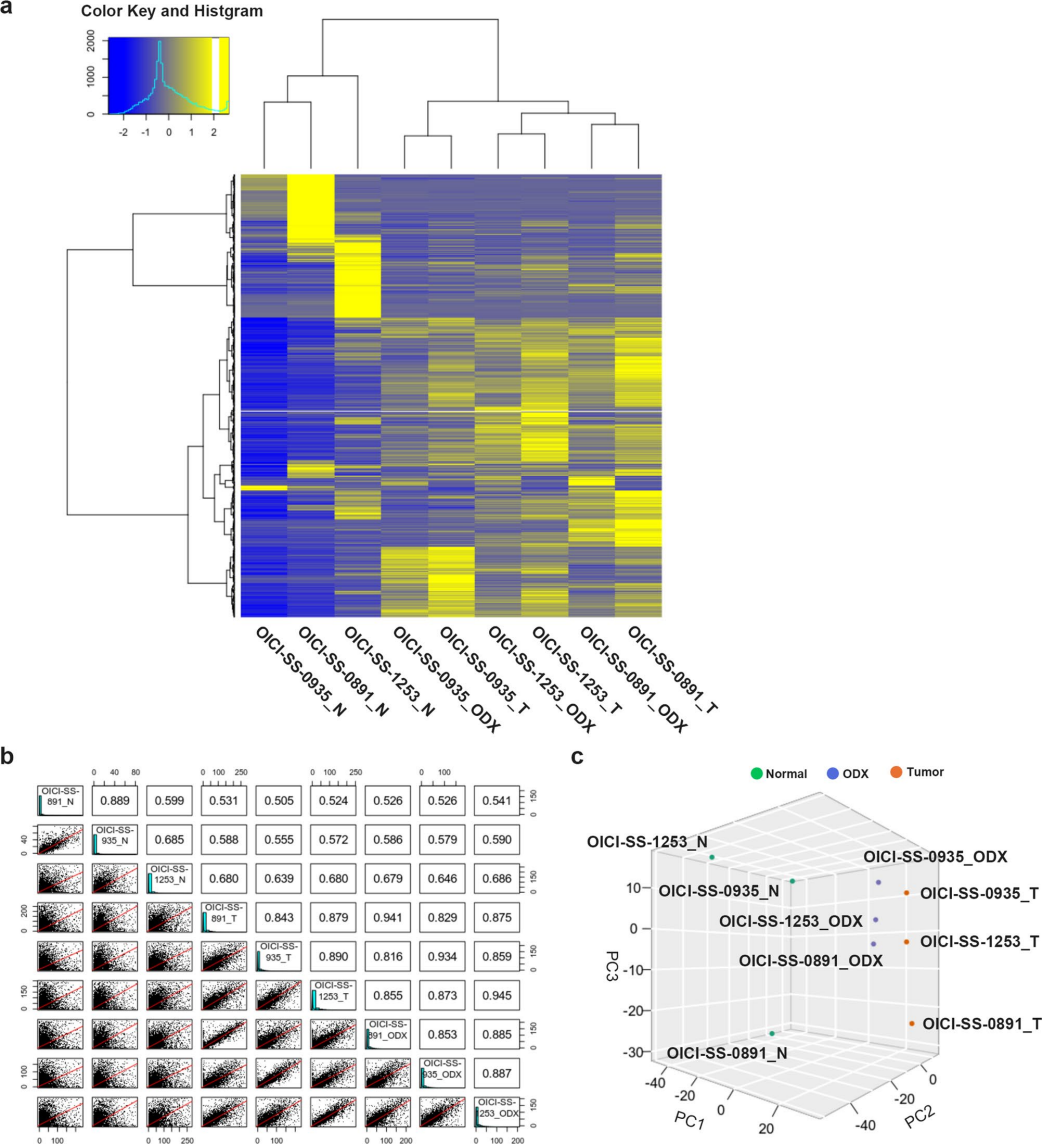
Comparison of the characteristics of SS organoids and original tumors through transcriptome analysis. (**a**) Heatmap showing the gene expression profiling (2466 genes) of normal tissue (N: Normal), original SS tumor (T: Tumor), and SS ODX tumors developed in NSG mice. (**b**) Correlation matrix of gene expression between SS ODX, original SS tumors, and normal tissues. R represents the correlation coefficient. (**c**) Principal component analysis (PCA) between Normal, original SS tumors, and ODX. ODX, organoid-derived xenograft; SS, synovial sarcoma.

We performed RNA-seq of paired tumor and normal samples from patients with SS, and developed tumors in mice. Through cluster analysis of RNA-seq data visualized with a heatmap, we observed that each pair of original tumors and ODX grouped together in the same cluster (Fig. 4a). Correlation analysis also demonstrated that the original tumors and ODX of each pair were strongly correlated (Fig. 4b). Principal component analysis showed a similar trend between the original tumors and ODX of each pair (Fig. 4c). These results suggest that gene expression signatures were preserved between primary SS tumors and ODX.

Transcriptome analysis showed that 9734 and 1643 genes were upregulated and downregulated, respectively, in the original SS tumor and ODX compared with those in normal tissue at fold changes > 2 (Supplementary Fig. S2a and Tables S2). We performed Kmeans Enrichment analyses for functional and ontological characterization of the genes (Supplementary Fig. S2b and Tables S3 and S4). Gene expression was clustered into four groups, and cluster B was enriched in both the original SS tumors and ODX. In this cluster, 1149 genes and 15 biological pathways of KEGG dataset were identified, including the signaling pathways regulating stem cell pluripotency (22 genes), the Wnt signaling pathway (24 genes), and the Hippo signaling pathway (26 genes) (Supplementary Fig. S2b and Table S4)^32–34^. In contrast, clusters C (275 genes and 15 biological pathways) and D (244 genes and 8 biological pathways) exhibited low expression levels in tumors, which included various metabolic pathways (Supplementary Fig. S2b and Table S4).

### SS organoids exhibited ferroptosis resistance despite low *ME1* expression

**Fig. 5:**
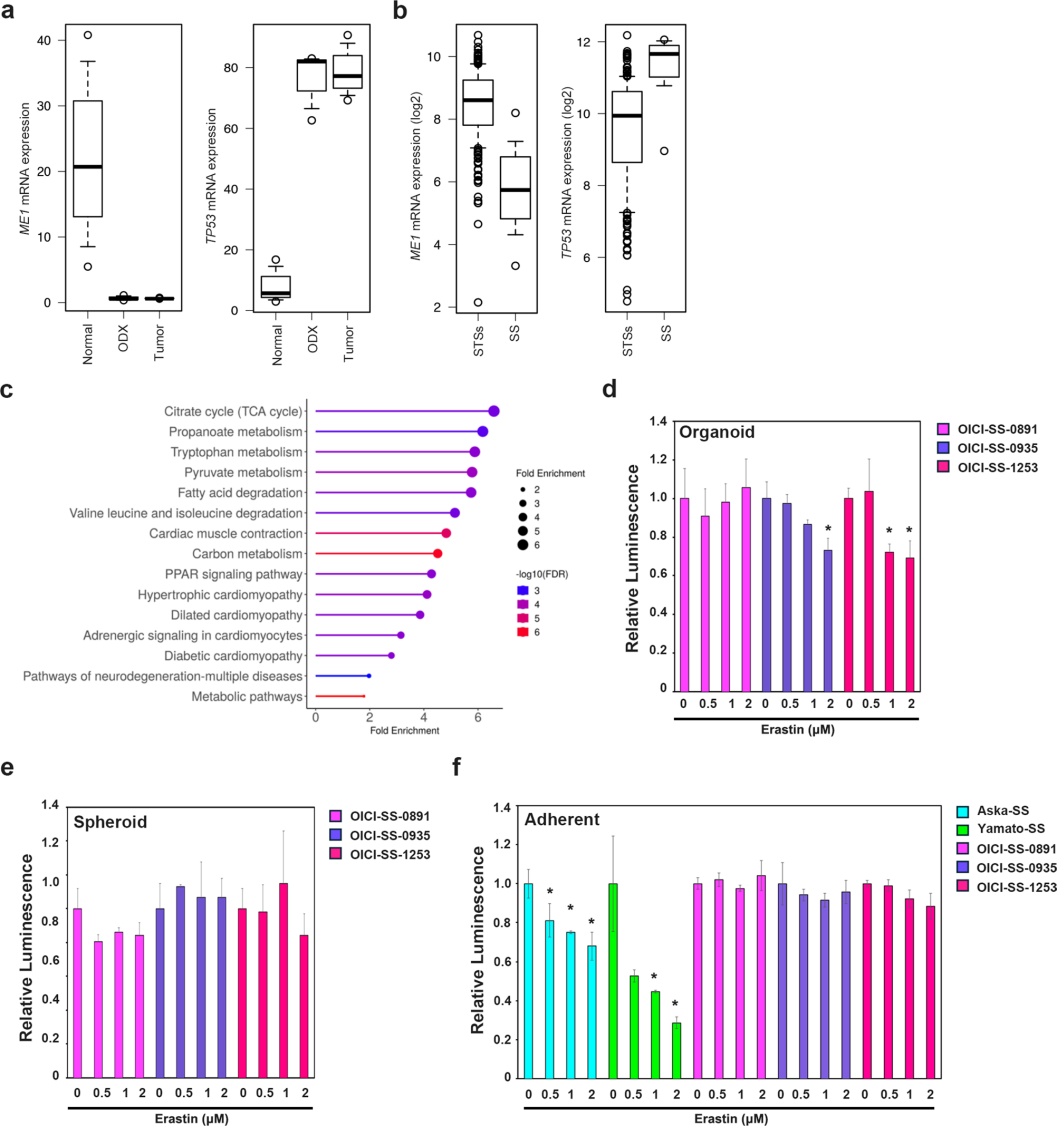
*ME1* expression and drug efficacy experiments against ferroptosis-inducing agents in SS organoids. (**a**) Comparison of *ME1* and *TP53* expression between SS original tumors, SS organoids and normal tissues based on RNA-seq data. (**b**) Comparison of *ME1* and *TP53* expression in SS relative to that in other malignant soft tissue tumors based on TCGA data. (**c**) Enrichment analysis using RNA-seq data revealed the accumulation of multiple metabolic pathways. The enrichment analysis represented pathways altered in both patient tumors and corresponding ODX. Pathway image generated using KEGG software; reproduced under permission of Kanehisa Laboratories. (**d**) Cell viability of SS organoids treated with erastin for 3 days (n = 3; *P < 0.05). Asterisks indicate statistically significant differences compared with controls. (**e**) Cell viability of SS spheroids treated with erastin for 3 days (n = 3; *P < 0.05). (**f**) Cell viability of SS cells cultured in adherent conditions treated with erastin for 3 days (n = 3; *P < 0.05). Drug response analyses of (**d–f**) were conducted using ODX-derived organoids. Yamato-SS and Aska-SS cells were used for positive control for erastin treatment experiments. SS, synovial sarcoma.

To conduct drug sensitivity experiments on established SS organoids, we focused on recent reports highlighting the high sensitivity of SS to ferroptosis-inducing agents^35,36^. SS organoids exhibited minimal *ME1* expression compared with that of normal tissue (muscle tissue) (Fig. 5a). Additionally, low *ME1* expression is associated with elevated *TP53* expression, and this phenomenon was replicated in SS organoids (Fig. 5a)^37^. Data from numerous clinical samples from cBioPortal also demonstrated a lack of *ME1* expression and its inverse correlation with *TP53* expression in SS (Fig. 5b). Various metabolic pathways are disrupted in SS and enrichment analysis revealed similar abnormalities accumulated in SS organoids (Fig. 5c)^32–34^. These findings indicate that the established SS organoids retained key factors regulating the response to ferroptosis, consistent with those observed in clinical SS tumors.

Based on these findings, we predicted that SS organoids would exhibit high sensitivity to the ferroptosis-inducing agent, erastin. Contrary to our expectations, erastin treatment resulted in a modest reduction in organoid viability (Fig. 5d). We hypothesized that these results were due to differences in culture conditions. When erastin was added to spheroid culture conditions, no significant growth inhibition was observed (Fig. 5e). The same drug experiments were conducted under adherent culture conditions. However, when the SS organoids were transitioned to adherent culture, they exhibited insufficient growth inhibition compared with that of the conventionally established SS cell lines, Yamato-SS and Aska-SS (Fig. 5f). These results demonstrated that SS organoids were resistant to ferroptosis-inducing agents, regardless of culture conditions.

## Discussion

This study represents the first report of highly accessible models, along with an efficient method for establishing organoids in SS. Our research group previously reported organoid models of epithelioid sarcoma and malignant giant cell tumors of the bone using the ALI organoid culture method^26,27^. In these cases, a medium developed for prostate cancer was used. However, to establish SS, a stem cell culture medium was used. We also established organoid cultures for various other histological subtypes of sarcoma using a stem cell culture medium and observed that the sarcoma cells exhibited relatively enhanced viability compared to that with previous methods. Consequently, the success rate of sarcoma organoid tumor formation in mouse xenografts increased to approximately 40%. We consider that the current version of the sarcoma organoid culture method is highly versatile, as it can be efficiently established, even for the most frequently occurring histological subtypes of sarcoma, including osteosarcoma, chondrosarcoma, liposarcoma, and leiomyosarcoma.

Brashears et al. demonstrated that SS lacked *ME1* expression in human tumor samples, using an RNA microarray of metabolic enzymes^35^. They also showed that *ME1* absence sensitizes SS cells to erastin-induced ferroptotic cell death. Tamiya et al. also reported that ferroptosis may have a therapeutic effect on SS^36^. All SS organoids in this study lacked *ME1* expression. The absence of *ME1* expression was reciprocally associated with the upregulation of *TP53* expression, as previously reported^37^. Therefore, we hypothesized that ferroptosis-inducing agents would be highly effective in SS organoids. However, contrary to our expectations, SS organoids exhibited a limited response to the agent. Therefore, we hypothesized that drug resistance was induced by the three-dimensional culture conditions. It has been frequently reported that tumor cells in three-dimensional cultures often exhibit higher drug resistance compared with those in adherent cultures^38–41^. Contrary to expectations, SS exhibited resistance to ferroptosis-inducing agents not only under three-dimensional culture conditions, such as organoids and spheroids, but also in adherent cultures. These results indicate that the established organoids exhibit distinct properties compared with conventionally established cell lines, potentially owing to differences in selection bias and cellular heterogeneity. The organoids also exhibited a certain proliferative capacity even under conditions of reduced *SS18-SSX* fusion gene expression. This finding suggests that chemotherapy administered prior to organoid establishment may have selected for cell populations that are less dependent on the fusion gene for growth. Consequently, the response to ferroptosis inducers might also have been affected by prior treatment. Notably, OICI-SS-0891, which had the longest treatment history, showed the greatest resistance to erastin. This clone also demonstrated the slowest proliferation rate, which could partly explain why suppression of the fusion gene and sensitivity to ferroptosis-inducing drugs were less evident in this model.

Given that the drug responsiveness remains relatively similar when transitioning from an organoid to an adherent culture, this approach could potentially enable high-throughput drug screening^42,43^. The ALI method is difficult to scale down for high-throughput drug screening. To establish this method for drug testing, it is necessary to validate similarities in drug efficacy between adherent cultures and organoid models using multiple organoids and various tissue types. If this validation is achieved, sarcoma organoids have the potential to become powerful tools for the development of novel therapies, not only for sarcomas, but also for various other cancer types.

The present study had several limitations. First, we established SS organoids using the ALI organoid culture method at a single institution. Therefore, whether ALI is a highly reproducible culture technique for sarcoma organoids remains unclear. Second, this study was conducted at a single institution, which may have introduced unintentional bias in the experimental procedures. Therefore, these potential biases cannot be eliminated. Further investigations using a larger cohort of sarcoma samples are necessary to validate our findings.

In conclusion, the newly established SS organoid line preserved the morphological and genetic characteristics of the original SS tumors. Established SS organoids exhibited tumorigenesis in NSG mice; thus, they are an excellent bioresource for further investigation of the pathogenesis and novel treatments of SS. This study demonstrates that SS organoids exhibit distinct characteristics compared to conventional cell lines and could serve as a valuable tool for exploring novel therapeutic strategies.

## Methods

### Patients

We treated five patients diagnosed with SS in our hospital in 2022–2023. All patients were Japanese, and three were male and two were female. They were middle-aged and approximately 40–60 years old at the time of tumor sample collection. After obtaining informed consent, we attempted to establish organoids from residual tumor samples obtained during surgery. Clinical information and detailed ODX data were summarized in Table 1, and the detailed clinical courses were described in Supplementary Fig. S1. Written informed consent was obtained from all participants. This study was approved by the Ethics Committee of Osaka International Cancer Institute (approval number: 1710059174-12), and all procedures were conducted in accordance with institutional and national ethical guidelines.

### Culture medium

The basal medium consisted of Advanced DMEM/F12 (Thermo Fisher Scientific, Waltham, MA, USA) supplemented with HEPES (10 mM, Thermo Fisher Scientific), GlutaMAX (1×, Thermo Fisher Scientific), and penicillin–streptomycin-glutamine (1×, Thermo Fisher Scientific).

The StemFit culture medium (AK02N; Ajinomoto Healthy Supply, Tokyo, Japan) was used as the organoid culture medium. The medium was prepared according to manufacturer’s instructions. First, for the primary culture of Case 1 (OICI-SS-0891), we used an organoid culture medium for prostate cancer as previously described^22,23^. However, after culturing the developing tumors in mice, we changed the StemFit medium because the culture conditions were favorable and stable.

### Establishment of novel PDO and ODX from human SS

Primary SS tumor tissues were obtained and prepared from the remaining surgically resected tumors as previously described^22,23^. After washing with ice-cold phosphate-buffered saline, tumor fragments were minced with sterile scissors and digested with 50 μg/mL Liberase TH (Sigma-Aldrich) for 15 min at 37 °C. The digestion was stopped by incubation in fetal bovine serum (FBS) for 15 min at 37 °C, followed by two washes with DMEM (Gibco). The cell suspension was filtered through a 1000-μm cell strainer (pluriSelect) to remove debris and resuspended in basal medium. Prepared cells were embedded in a collagen type I gel (Cellmatrix type I-A, Nitta Gelatin Inc.) and cultured at 37 °C in 5% CO₂ and 100% humidity using an ALI culture system. The organoid medium (1.5 mL) was replaced every 3 days. For passaging, both the upper and lower collagen layers containing organoids were minced and re-embedded in fresh collagen following the same procedure. After serial passaging with ALI organoid cultures, the SS organoids were xenografted into 6-week-old NSG mice. Following tumor development, the tumors were resected from the mice and subjected to the tissue preparation steps described above^22,23^. Serial passages and xenografts in NSG mice were repeated for ALI organoid cultures. Upon tumor development, PDO and ODX were established using SS. The mouse experiments were conducted as previously described^22,23^. The tumors were subsequently resected and used in various experiments as described below. Animals were monitored daily for health status. Body weight was measured only when abnormal appearance or behavior suggested potential illness; however, no animals showed signs of illness or weight loss during this study. Tumor size was monitored by caliper measurements, and tumor weights were not obtained at necropsy. Mice were anesthetized with isoflurane and euthanized by cervical dislocation under deep anesthesia. All procedures were performed in compliance with institutional animal care guidelines. The study was conducted in compliance with the ARRIVE guidelines. The animal experiment was approved by the Animal Experiment Committee of Osaka International Cancer Institute (approval number: 25030321), and all procedures were carried out in accordance with the relevant guidelines and regulations.

### RNA isolation and RT-PCR

Total RNA was extracted using the RNeasy Plus Universal Mini Kit (Qiagen, Hilden, Germany) following the manufacturer’s protocol and purity was assessed using the A260/A280 ratio. Superscript IV VILO (Invitrogen, Carlsbad, CA, USA) was used for cDNA synthesis according to the manufacturer’s instructions. RT-PCR was performed using the PrimeSTAR MAX DNA polymerase (Takara, Shiga, Japan). The primers designed for *SS18-SSX1* and *SS18-SSX2* are listed on Supplementary Table S1.

### DNA isolation

Total DNA was extracted using a DNeasy Blood & Tissue Kit (Qiagen) according to the manufacturer’s protocol.

### Sequence analysis

*SS18-SSX* cDNA was identified using PCR with a primer set for *SS18-SSX1* or *SS18-SSX2* (Supplementary Table S1). For sequence analysis, the RT-PCR-amplified *SS18-SSX* cDNA fragments were analyzed on 1.0% agarose gels, purified using a Qiagen gel extraction kit (Qiagen) and sequenced by Eurofins Genomics with the forward or reverse primers mentioned above. BLAST software (http://blast.ncbi. nlm.nih.gov/Blast.cgi) was used for computer analysis of sequence data.

### Immunohistochemistry

Immunohistochemical studies were conducted to determine whether the phenotypes of the cultured organoids and developed tumors corresponded to those of the original tumors, as previously described^41^. Primary antibodies against SS18-SSX (E9X9V) XP (1:500; Cell Signaling Technology, Danvers, MA, USA) were used.

### Western blotting

The following antibodies were used for western blotting: anti-SS18-SSX (E9X9V) XP (1:1000, Cell Signaling Technology) and anti-vinculin (7F9) (1:1000; Santa Cruz Biotechnology, CA, USA). Horseradish peroxidase-conjugated anti-mouse IgG (#115–035-146, 1:2000) and antirabbit IgG (#711–035-152, 1:2000) were purchased from Jackson ImmunoResearch. Cell lysates of Yamato, SYO1, and HSSY2 were provided by Research Institute Nozaki Tokushukai Hospital and used in this study. Western blotting was performed according to a previously described standard protocol^41^. The gel shown in the figure was derived from a single experiment. The upper half was re-exposed to optimize visualization and used as a separate panel. A dividing space has been added for clarity. The full-length, uncropped gel is provided in Supplementary Supplementary Fig. 4a.

### Quantitative RT-PCR analysis

Total RNA was extracted from the cells using TRIzol reagent and reverse-transcribed into cDNA using SuperScript IV VILO (Thermo Fisher Scientific) according to the manufacturer’s instructions. Quantitative RT-PCR was performed using TB Green Premix Ex Taq II (TaKaRa Bio) and gene-specific primer pairs on a CFX96 Touch Real-time PCR system (Bio-Rad Laboratories, Hercules, CA, USA). Gene-specific primer sequences (Supplementary Table S1) were obtained from the PrimerBank database (https://pga.mgh.harvard.edu/primerbank/index.html). *TBP* was used as an internal standard.

#### Cell proliferation assay

For adherent culture, we prepared collagen I-coated 96-well plates (Iwaki; AGC TECHNO GLASS Co., Ltd., Shizuoka, Japan). The cell suspension was prepared according to the organoid passage or tissue preparation procedures. Cell suspensions were prepared from the SS organoids. The cells were spread onto plates containing the culture medium and cultured in a CO_2_ incubator. The culture medium was replaced with an elastin-containing medium 24 h later and cells were cultured for either 3 or 7 days. Cell viability was assessed using the Cell Titer-Glo assay. During the growth curve experiments, the initial cell numbers were set to 800, 800, and 1000 for OICI-SS-891, OICI-SS-935, and OICI-SS-1253, respectively. In the erastin drug experiment, the initial cell number was set at 2000 cells for all three SS organoids. Yamato-SS and Aska-SS cells were passaged two to three times in stem cell culture medium prior to the drug experiment to ensure stable proliferation before use.

For spherical culture, we prepared ultra-low attached dishes and PrimeSurface® 96U plate. The cell suspension was prepared according to the organoid passage or tissue preparation procedures. The cell suspensions (400 cells) were spread onto dishes or plates containing the culture medium and cultured in a CO_2_ incubator. The culture medium was replaced with elastin- or lentivirus-containing medium 24 h later, and the cells were cultured for 3 days. Total RNA was collected and cell viability was assessed using the Cell Titer-Glo assay.

#### Organoid proliferation assay

Cultured SS organoids or tumors developed in mice were used for the proliferation assays. The cell suspension was prepared according to the organoid passage or tissue preparation procedures. The collagen gel matrix was prepared according to an organoid culture procedure. To prepare the culture dishes, Millicell culture plate inserts (PICM 01,250, Millicell-CM, Millipore) were placed in a culture plate with 24 wells (Thermo Fisher Scientific). In this experiment, collagen-based monolayer culture was used for cell cultivation. After the tissue preparation steps described above, the prepared cells were counted and cultured in the inserts of the prepared 300 μl of collagen with 10 μl of organoid culture medium at 37 °C with 95% air, 5% CO2, and 100% humidity. The cell seeding density was set at 1 × 10^4^ cells for well. The culture medium was replaced with an erastin-containing medium 72 h later, and the cells were cultured for either 3 or 7 days. Cell viability was assessed using the Cell Titer-Glo assay.

#### Lentivirus generation and infection

The lentiviral packaging mixtures (pLP1, pLP2, and pLP/VSVG) were purchased from Invitrogen. SS18-SSX shRNA was provided by Research Institute Nozaki Tokushukai Hospital. The target sequence oligos (Supplementary Table S1) were annealed and inserted into the pLKO.1 puro plasmid according to the protocol provided by Addgene. The GFP expression vector, pLentiCMV GFP Puro (658-5), or SS18-SSX shRNA plasmid and lentivirus packaging mixture were co-transfected into 293FT cells using PEI-MAX instead of Lipofectamine 2000, according to the manufacturer’s instructions. Lentivirus generation and infection were performed as previously described^22,41^.

#### RNAseq

Total RNA samples were sent to Seibutsu Giken Inc. (Kanagawa, Japan) for next-generation sequencing. RNA concentrations were measured using a Quantus Fluorometer and the Quanti Fluor RNA System (Promega). RNA integrity was assessed using the 5200 Fragment Analyzer System and the Agilent High Sensitivity RNA Kit (Agilent Technologies). RNA sequencing libraries were prepared using the MGIEasy RNA Directional Library Prep Set (MGI Tech Co., Ltd.) according to the manufacturer’s protocol. The concentration of the prepared libraries was measured using a Qubit 3.0 Fluorometer and the dsDNA HS Assay Kit (Thermo Fisher Scientific). Library quality was evaluated using the Agilent 2100 Bioanalyzer with the High Sensitivity DNA Kit, as well as the Fragment Analyzer with the dsDNA 915 Reagent Kit (Agilent Technologies). Circular DNA was generated from the libraries using the MGIEasy Circularization Kit (MGI Tech Co., Ltd.). DNA nanoballs (DNBs) were produced using the DNBSEQ-G400RS High-throughput Sequencing Kit (MGI Tech Co., Ltd.) according to the manufacturer’s instructions. Paired-end sequencing (2 × 100 bp) was performed on the DNBSEQ-G400 or DNBSEQ-T7 platform. Adapters were trimmed from the raw FASTQ files using Cutadapt (v4.0), and low-quality reads (quality score < 20) as well as paired-end reads shorter than 75 bases were filtered out using Sickle (v1.33). Read alignment to the GRCh38 human reference genome was performed using HISAT2 (v2.2.1). The resulting SAM and BAM files were processed using SAMtools for sorting and indexing. Gene-level read counts were calculated using featureCounts (v2.0.3). Gene expression levels were normalized using reads per kilobase per million mapped reads (RPKM) and transcripts per million (TPM). Matched normal skeletal muscle tissue was used as the normal control for the transcriptomic analysis. Genetic characteristics and transcriptomic profiles were analyzed and visualized using the tools include RIAS (Rhelixa Integrated Analyzers, version not specified; https://rias.rhelixa.com/rias, Rhelixa Co., Ltd., Tokyo, Japan), iDEP (ver.951, http://bioinformatics.sdstate.edu/idep95/), and RaNA-seq (version not specified; https://ranaseq.eu/)^45,46^.

#### TCGA data

Gene expression data for *ME1* and *TP53* in patients with soft tissue sarcoma were collected from the TCGA database (Sarcoma and Firehose Legacy).

#### Statistical analysis

Statistical significance was set at *P* < 0.05. Statistical analyses were performed using Student’s t-test in Microsoft Excel (Microsoft). Ethical approval was obtained from the institutional review board of Osaka International Cancer Institute.

## Supplementary Information

Supplementary Material 1

Supplementary Material 2

Supplementary Material 3

Supplementary Material 4

Supplementary Material 5

Supplementary Material 6

## Data Availability

All data generated or analyzed during this study, except for the RNA sequencing data, are included in this published article and its supplementary information files. The RNA-seq datasets generated and/or analyzed during the current study are available in the DDBJ Sequence Read Archive (DRA) under the accession number JGAS000806: https://humandbs.dbcls.jp/en/hum0401-v3
