# Supplementary material for: Development of synovial sarcoma organoids exhibiting ferroptosis resistance despite low malic enzyme 1 expression
Source: Sci Rep. 2025 Dec 21;16:2369. doi: 10.1038/s41598-025-32030-w (PMC12816075; doi:10.1038/s41598-025-32030-w)

**Supplementary information**

**Supplementary Fig. 1. (a)** Case1; The patient was a 42-year-old female who presented with a tumor approximately 5 cm in size beneath the right pectoralis major muscle. Tumor resection and chest wall resection were performed, leading to a diagnosis of synovial sarcoma (SS18-SSX positive). Subsequently, local recurrences and multiple lung metastases occurred, necessitating tumor resection and partial lung resection. Systemic therapy with doxorubicin (DOX) and ifosfamide (IFM) was initiated. One year later, progressive disease (PD) was confirmed by CT, prompting a switch to pazopanib. After another year, disease progression was observed, and the treatment regimen was changed to trabectedin. Long-term disease control was subsequently achieved through concurrent radiofrequency ablation (RFA). A tumor sample for organoid establishment was obtained from a recurrent lesion in the right chest wall approximately ten years after the initial diagnosis. The patient continues treatment with trabectedin and pazopanib.

**(b)** Case2; The patient was a 50-year-old Japanese man. The primary tumor was located in the dorsal soft tissue of the left hand, and surgical resection was performed, leading to a diagnosis of synovial sarcoma. Adjuvant chemoradiotherapy (CRT) was administered. One year later, lung metastases were detected, and partial lung resection was performed, followed by postoperative chemotherapy with IFM. Five years later, the patient experienced local recurrence, which was detected via MRI due to new-onset pain in the left hand, leading to finger amputation surgery. Subsequently, high-dose ifosfamide (HD-IFM) was initiated. However, 15 years after the initial diagnosis, metastases to the right lung and pleural dissemination were observed, prompting right lung segment resection. During the eighth cycle of HD-IFM, the patient developed severe encephalopathy. Despite switching to pazopanib, disease progression continued, and best supportive care (BSC) was provided. A tumor sample for organoid establishment was obtained from a metastatic lesion in the right lung approximately 15 years after the initial diagnosis.

**(c)** Case3; The patient was a 66-year-old female who developed swelling in the right distal thigh two years prior and was receiving treatment at a local orthopedic clinic. As the knee joint swelling progressed, MRI revealed a tumor, and a needle biopsy confirmed the diagnosis of synovial sarcoma. While no distant metastases were detected, the tumor involved nerves and blood vessels. Following joint aspiration, a

femoral amputation was performed. Eight months later, pulmonary metastases emerged and exhibited a progressive trend. Consequently, approximately one year after the initial surgery, a pulmonary metastasectomy was performed. The patient is currently under follow-up with no evidence of disease (NED). A tumor sample for organoid establishment was obtained from a primary lesion in the right thigh.

**Supplementary Fig. 2. (a)** Volcano plot of differentially expressed genes in SS tumors and ODXs compared to normal tissue. **(b)** Heatmap of Kmeans Enrichment analyses in Normal, SS tumors, and ODXs. Pathway image generated using KEGG software; reproduced under permission of Kanehisa Laboratories.

**Supplementary Fig. 3. (a)** Cell viability of SS organoids treated with erastin for 7days (n = 3; *P < 0.05). **(b)** Cell viability of SS cells cultured adherent condition treated with erastin for 7days (n = 3; *P < 0.05). Yamato-SS and Aska-SS cells were used for positive control for Erastin treatment experiments.

**Supplementary Fig. 4.** Uncropped PCR and Western blot images related to Fig. 3. (a) Uncropped PCR gel images showing *SS18-SSX* fusion transcripts in the three synovial sarcoma organoid lines corresponding to Fig. 3a-c. (b) Uncropped PCR gels showing *GAPDH* as an experimental control corresponding to Fig. 3a-c. (c) Uncropped Western blot images for SS18-SSX shown under three exposure conditions (short, medium, and long exposure), corresponding to Fig. 3d. (d) Uncropped Western blot for Vinculin corresponding to Fig. 3d. Only one exposure was obtained during the original experiment; therefore, no additional exposure conditions are available. All images were obtained from the original experimental runs without cropping or digital processing. Protein loading was quantified prior to electrophoresis, and 30 μg of total protein was loaded per lane. The Western blot in (c) and (d) was derived from a single gel; the upper portion was re-exposed to optimize visualization and is shown as separate panels in the main figure. A dividing space has been added for clarity. Full-length, uncropped images are provided here to comply with Nature Research digital image integrity policies.

**Supplementary Table S1.** List of primers and shRNA used in current study

**Supplementary Table S2.** Differentially expressed genes in SS tumors and ODXs compared to normal tissue

**Supplementary Table S3.** Genes of each cluster with Kmeans Enrichment analyses in Normal, SS tumors, and ODXs

**Supplementary Table S4.** KEGG pathways of each cluster with Kmeans Enrichment analyses in Normal, SS tumors and ODXs.

**Supplementary Table S5.** Raw data from experiments other than RNA sequencing conducted in this study, including cell proliferation assays, drug response evaluations, qPCR results and TCGA data. These data support the findings presented in the main figures.

# Supplementary Figure 1

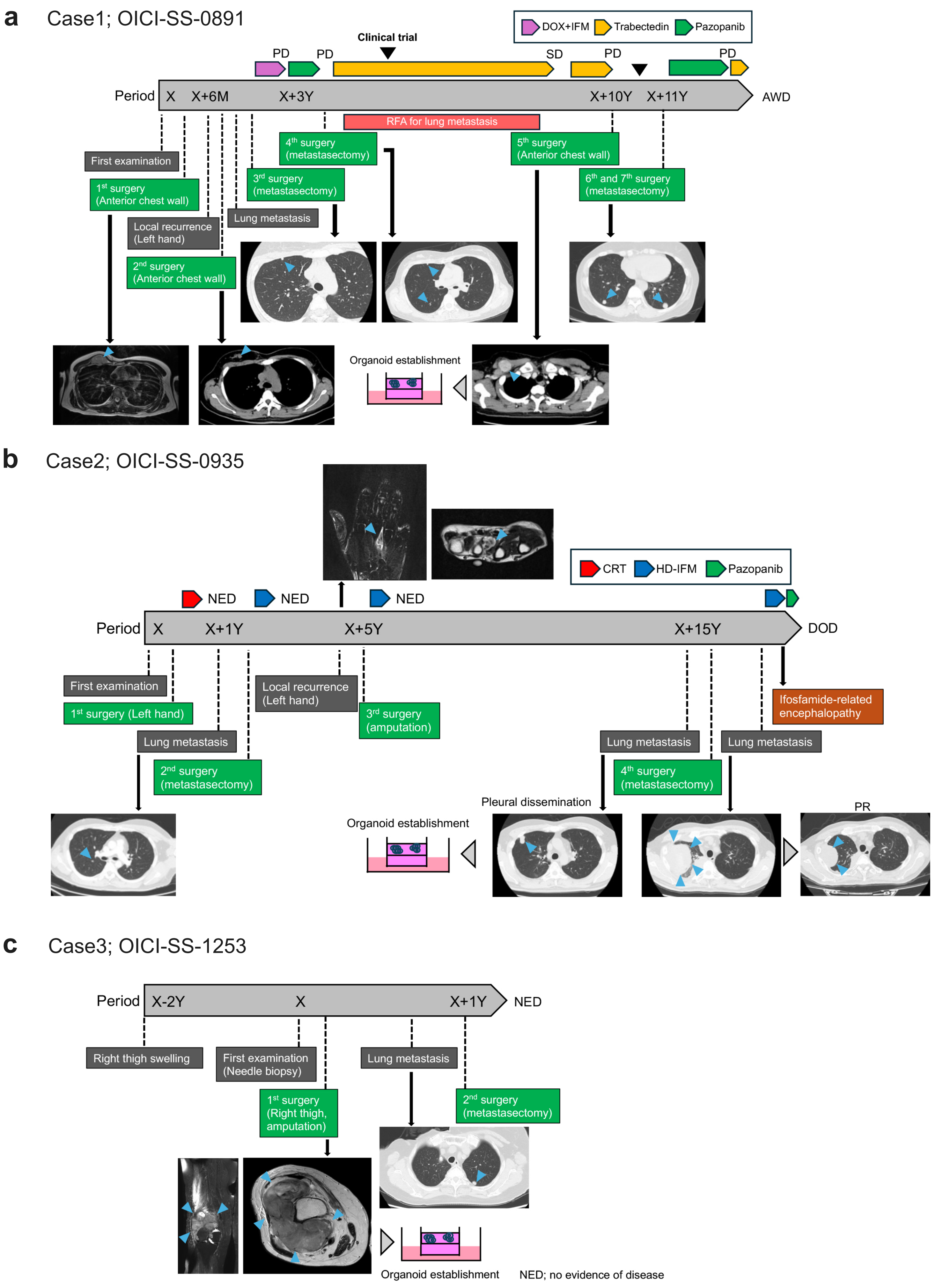

# Supplementary Figure 2

a

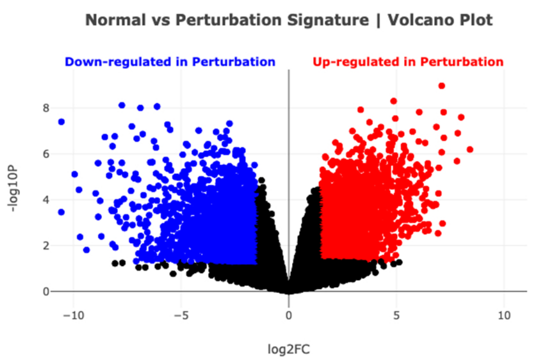


b

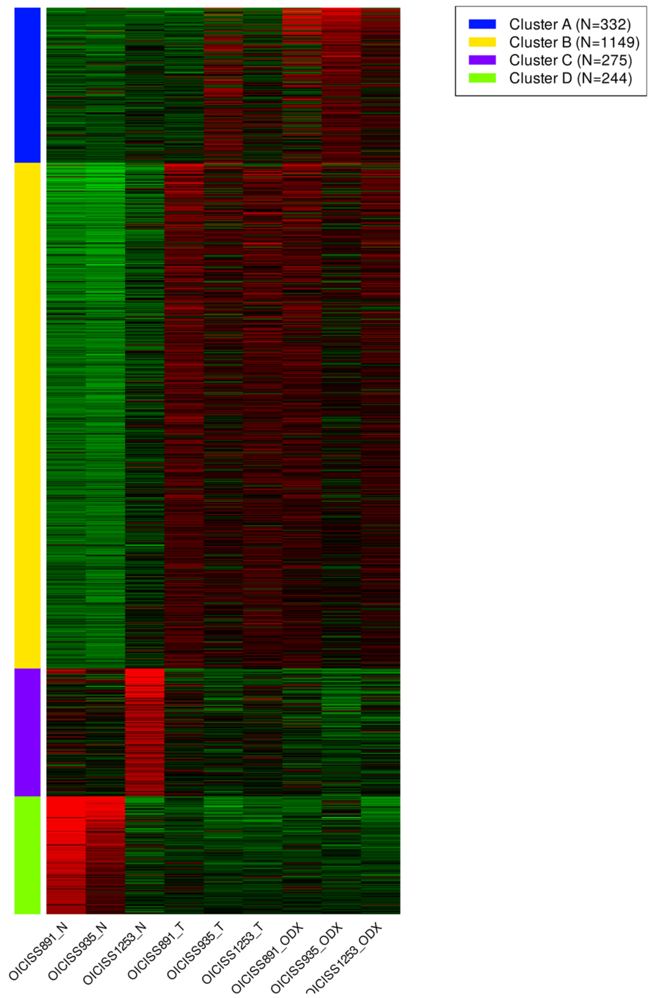

# Supplementary Figure 3

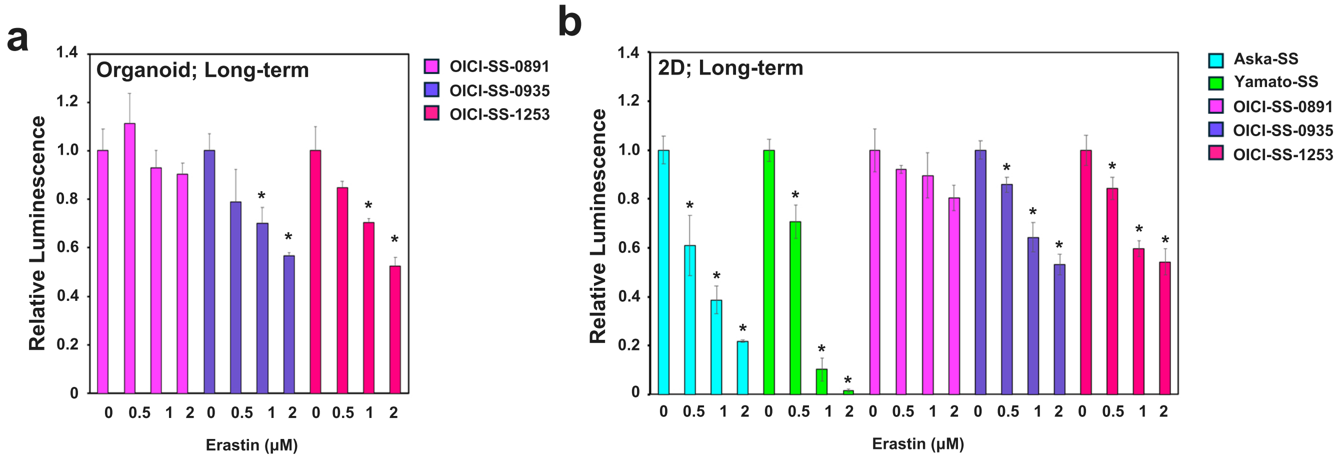

# Supplementary Figure 4

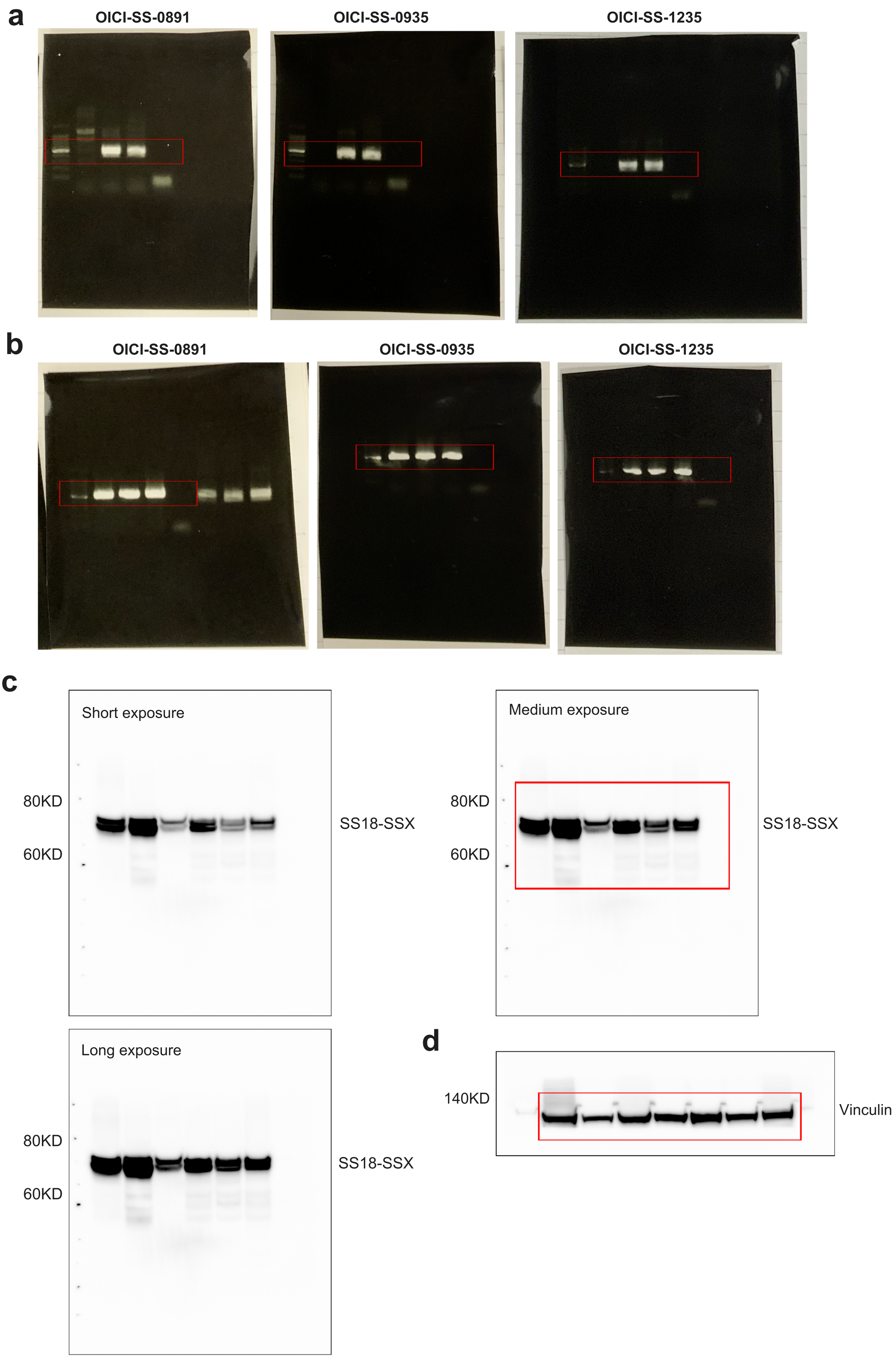

Supplement: Supplementary file 6 — Supplementary Material 6 [file 41598_2025_32030_MOESM6_ESM.pdf]
